# Improving the safety and experience of transitions from hospital to home: a cluster randomised controlled feasibility trial of the 'Your Care Needs You' intervention versus usual care

**DOI:** 10.1186/s40814-022-01180-3

**Published:** 2022-10-01

**Authors:** Ruth Baxter, Jenni Murray, Sarah Cockayne, Kalpita Baird, Laura Mandefield, Thomas Mills, Rebecca Lawton, Catherine Hewitt, Gerry Richardson, Laura Sheard, Jane K. O’Hara, on behalf of the PACT research team

**Affiliations:** 1 Yorkshire Quality and Safety Research Group, Bradford Institute for Health Research, Bradford, UK; 2 School of Psychology, University of Leeds, Leeds, UK; 3 York Trials Unit, University of York, York, UK; 4 Centre for Health Economics, University of York, York, UK; 5 School of Healthcare, University of Leeds, Leeds, UK

**Keywords:** Transitions of care, Hospital discharge, Cluster randomised controlled trial, Feasibility trial, Complex intervention, Patient safety, Patient experience, Care of older people

## Abstract

**Background:**

The ‘Your Care Needs You’ (YCNY) intervention aims to increase the safety and experience of transitions for older people through greater patient involvement during the hospital stay.

**Methods:**

A cluster randomised controlled feasibility trial was conducted on NHS inpatient wards (clusters) where ≥ 40% of patients were routinely ≥ 75 years. Wards were randomised to YCNY or usual care using an unequal allocation ratio (3:2). We aimed to recruit up to 20 patients per ward. Follow-up included routine data collection and questionnaires at 5-, 30-, and 90-days post-discharge. Eligible patients were ≥ 75 years, discharged home, stayed overnight on participating wards, and could read and understand English.

The trial assessed the feasibility of delivering YCNY and the trial methodology through recruitment rates, outcome completion rates, and a qualitative evaluation. The accuracy of using routinely coded data for the primary outcome in the definitive trial was assessed by extracting discharge information for up to ten nonindividual consenting patients per ward.

**Results:**

Ten wards were randomised (6 intervention, 4 control). One ward withdrew, and two wards were unable to deliver the intervention. Seven-hundred twenty-one patients were successfully screened, and 161 were recruited (95 intervention, 66 control). The patient post-discharge attrition rate was 17.4% (*n* = 28). Primary outcome data were gathered for 91.9% of participants with 75.2% and 59.0% providing secondary outcome data at 5 and 30 days post-discharge respectively. Item completion within questionnaires was generally high. Post-discharge follow-up was terminated early due to the COVID-19 pandemic affecting 90-day response rates (16.8%). Data from 88 nonindividual consenting patients identified an error rate of 15% when using routinely coded data for the primary outcome. No unexpected serious adverse events were identified.

Most patients viewed YCNY favourably. Staff agreed with it in principle, but ward pressures and organisational contexts hampered implementation. There was a need to sustain engagement, provide clarity on roles and responsibilities, and account for fluctuations in patients’ health, capacity, and preferences.

**Conclusions:**

If implementation challenges can be overcome, YCNY represents a step towards involving older people as partners in their care to improve the safety and experience of their transitions from hospital to home.

**Trial registration:**

ISRCTN: 51154948.

**Supplementary Information:**

The online version contains supplementary material available at 10.1186/s40814-022-01180-3.

## Key messages regarding feasibility

- There is currently a lack of evidence regarding interventions that support the involvement of older people in their care during a hospital stay in order to improve safety and experience during the transition home.
- Results suggest a need to over recruit wards (clusters) and increase patient recruitment targets per ward to account for attrition. Although the intervention was generally considered acceptable, several implementation challenges were identified.
- The findings warrant proceeding to a definitive trial albeit with small methodological changes and an increased focus on senior manager buy-in and culture change to implement the intervention on wards.

## Background

Emergency hospital readmissions are rising in the UK [1]. In 2020/2021, 19.6% of older patients (aged 75 years and over) were readmitted within 30 days [1], around 30% of which are considered avoidable [2–5]. Up to 20% of patients experience an adverse event (e.g. medication error) during the transition from hospital to home [6], and, more broadly, patients report poor experiences [7–9]. These problems are often exacerbated in older patients due to complex care needs [10, 11] and greater service reliance [12].

The complex nature of transition interventions makes it difficult to decipher their effective components; however, evidence suggests successful interventions both educate and involve patients [13, 14]. This aligns with patients’ central role across transitional care pathways and a view that they can bridge gaps in their care [15–17].

The Partners at Care Transitions (PACT) research programme aims to improve the safety and experience of older people as they transition from hospital to home by enhancing patient involvement. Through the programme's earlier research [18–21], we identified four-patient activities to target in our intervention:

- Managing health and wellbeing
- Managing medications
- Completing daily activities (e.g. mobilising)
- Appropriately escalating care

These four activities are those that patients often relinquish responsibility for on hospital admission and then take up at discharge.

Underpinned by Resilient Health Care [22], we developed a theory of change to guide intervention development. Our theory suggests that patients need to ‘practise’ being at home during their hospital stay to gain the knowledge and skills required to enact these activities post-discharge [20]. The Your Care Needs You (YCNY) intervention was codesigned and further developed to support patients and families to ‘reach in’ to the system when gaps and vulnerabilities arise, thereby enhancing system resilience. This paper reports a cluster randomised controlled feasibility trial (cRCT) of the YCNY intervention. The objectives were as follows:

1. Explore the feasibility of methods to screen, recruit, retain, and follow up participants in the trial.
2. Determine the most accurate and feasible way of obtaining baseline, primary outcome (hospital emergency readmissions), secondary outcome, and health economic data.
3. Explore the acceptability, usefulness, and feasibility of the YCNY intervention components to patients, carers, and staff and to develop an implementation package via a qualitative evaluation.

## Method

### Trial design

A cluster randomised controlled feasibility trial was conducted on ten wards (clusters) randomised to the YCNY intervention or usual care. A cluster design was indicated due to ward-level implementation requiring support for culture and behaviour change. For consenting patients, outcome data were gathered from medical records and follow-up questionnaires administered at 5-, 30-, and 90-days post-discharge. A qualitative evaluation was undertaken involving observations and interviews.

YCNY supports transitions to patients’ *own homes*; however, the routine coding of NHS data does not distinguish between patients whose usual place of residence is their *own home* versus a *nursing/care home*. To calculate the potential ‘error rate’ that might arise from a reliance on coded discharge destination, we compared actual and coded discharge destinations in a sample of nonindividual consented patients. Permission was gained from the Confidentiality Advisory Group (CAG) to access confidential patient information without consent.

The protocol was published [23] and the trial registered on the International Standard Randomised Controlled Trials registry (ISRCTN: 51154948). The CONSORT (Consolidated Standards of Reporting Trials statement) guidelines for pilot and feasibility studies and TIDieR (Template for Intervention Description and Replication) checklist are provided in supplementary file 1 [24]. Ethical, CAG, and governance approvals were gained.

### Setting, clusters, and participants

Potential wards within three acute NHS trusts in England were identified using routine data and local intelligence. Eligible wards were NHS funded, inpatient wards where, in the preceding 12 months, at least 40% of patients were aged 75 years or over. Acute admission wards/units, wards without regular medical input, and those already participating in a trial were excluded.

Eligible patients were as follows:

- Aged 75 years and over
- Anticipated to be discharged to their own or relative’s home
- Staying overnight on a participating ward
- Able to read and understand English

Informal carers were recruited if patients lacked capacity. Patients were excluded if they lived out of area, were transferred to another acute hospital or nursing/residential home, and were admitted for psychiatric reasons or at the end of life.

Participants were screened and provided written consent to complete a baseline assessment, three post-discharge follow-up questionnaires, and for routine data to be extracted from their medical records. Participants received a £5 voucher with each follow-up questionnaire.

Eligible nonindividual consenting patients were aged 75 years and over and were coded as being discharged to their ‘usual place of residence’. On each participating ward, patients were consecutively sampled during the main recruitment period. Leaflets and posters were made available on all participating wards enabling patients to opt out of providing access to their routine data.

### Intervention

The development of YCNY is published elsewhere [20, 21]. YCNY consisted of three fixed, patient facing components:

i) A booklet encouraging patients and providing instructions on how to be more involved in their care in relation to the four activities (health, medicines, daily activities, and escalation). The booklet provided questions for patients to ask staff and could be propped up (e.g. on a bedside table) to act as a visual cue.
ii) A film introducing and emphasising the need for YCNY which targeted their beliefs about health and emotional consequences
iii) A patient-friendly care summary received on discharge providing practical and social support to patients post-discharge

Staff were encouraged to consider how they could support patients to use the booklet and respond to patient questions, thus providing social and emotional support. They were asked to explore how they currently support the four activities (health, medicines, daily activities, and escalation) and what they could do to enhance them, e.g. by encouraging patients to mobilise or to practice taking their own medications. The activities staff implemented to address these things were standardised by *function* (i.e. aim) rather than *component* (i.e. format) [25, 26] and could vary by ward. With fixed and variable components, YCNY can be considered a ‘hybrid’ intervention [27].

#### Implementation

We initially planned to implement YCNY in four stages [21]. First, a 1–2 h facilitation meeting enabled key multidisciplinary staff to tailor YCNY to their ward context and to identify a ‘coach’ (point of contact). Second, staff roles and responsibilities in supporting the intervention were decided (e.g. introducing the booklet and responding to patient questions). Third, brief training aimed to give staff the knowledge and skills required to deliver YCNY. Posters and handouts provided prompts and reminders. Finally, a ‘share and learn’ session, held shortly after wards started implementing YCNY, was designed to identify and resolve implementation problems.

#### Usual care

Control wards delivered usual care according to standard processes, policies, and procedures.

### Outcomes

#### Trial feasibility

Trial feasibility was assessed by screening, recruitment, retention, outcome completion rates, and conversations with staff and patients. The feasibility of conducting a full cost-effectiveness analysis was also assessed. Acceptability, usefulness, and feasibility of the intervention and implementation package were assessed by observations and interviews. Contamination was assessed through participants’ movements between control, intervention, and nonparticipating wards prior to discharge.

#### Routinely collected data

For consented participants, the following data were extracted from medical records at the end of the study:

- Emergency hospital readmission dates — to assess the feasibility of collecting primary outcome (30 days) and secondary outcome (60 and 90 days) data for a definitive RCT
- Ward moves during the index admission — to assess contamination and intervention fidelity

For nonindividual consenting patients coded as returning to their usual place of residence, actual discharge destinations were extracted from medical records and categorised as own/relative’s home, nursing/care home, intermediate care, or other. In addition, ward-level baseline data (readmission rates and average lengths of hospital stay) were gathered for the previous 12-month period (Oct 2018–September 2019).

Data were extracted from medical records by an employee of the participating NHS organisation. Before being and sent to the research team, consented participant data was pseudonymised, and nonindividual consented data was anonymised.

#### Participant reported baseline and follow-up data

Demographic information and three validated measures (Table 1) were collected at baseline, while the participant was in hospital. Follow-up was initiated at 5-, 30-, and 90-day post-discharge to align with the initial post-discharge period when safety problems more commonly occur, nationally reported emergency readmission data, and the longer-term transition period. Follow-up data were collected via a postal questionnaire and optional telephone call and included four validated measures (Table 1).

**Table 1:** Baseline and follow-up measurements

Validated measure	When	Description
**Patient at Care Transitions Measure (PACT — M)** [28]	5, 30, and 90 DPD	Assesses patient perceptions of the quality and safety of transitional care. Eight items are scored on a 5-point Likert scale from strongly disagree to strongly agree. Also measured are the incidences (yes or no) of seven adverse events post-discharge and associated details. Total scores range from 0 to 67
**EuroQol 5-Dimension Health Questionnaire (5 levels) (EQ-5D-5L) and proxy EQ-5D-5L** [29]	Baseline and 5, 30, and 90 DPD	Measures five quality-of-life dimensions which are scored on a 5-point Likert scale from no problems to unable. Scores can be used to generate quality-adjusted life years (QALYs). The measure also includes a visual analogue scale (1–100) to capture patients’ perceptions of their health. Informal carers completed the proxy EQ-5D-5L
**Care Transitions Measure 3 items (CTM-3)** [30]	5, 30, and 90 DPD	Measures patient perceptions of the quality of care transitions. Three items are scored on a 5-point Likert scale from strongly disagree to strongly agree. The CTM-3 scoring guide transforms scores onto a 0–100 scale
**Client Service Receipt Inventory (CSRI )** [31]	5, 30, and 90 DPD	Assesses patients’ use of health-related resources. Questions have been adapted to assess the health resources that are pertinent to care transitions from hospital to home for older people
**Utility of the YCNY intervention**	5 and 30 DPD	Non-validated questions about the receipt and usefulness of the intervention
**Functional Comorbidity Index (FCI)** [32]	Baseline	A sum of 18 self-reported comorbid conditions. A total score ranges from 0 to 18 with higher scores indicating greater comorbidity
**Barthel index (BI)** [33]	Baseline	10 items measuring activities of daily living and mobility. Scores range from 0 to 20, with lower scores indicating increased disability

*DPD* days post-discharge

### Sample size

Up to ten wards enabled basic statistical analyses with at least four clusters in each trial arm [34]. As this was a trial feasibility study, formal sample size calculations were not conducted [35, 36]. The trial aimed to recruit ten wards and 200 patients (20 per ward) to align with a recent cRCT [37] and to sufficiently assess trial feasibility. Up to 100 nonindividual consenting patients (ten per ward) were included in the study.

### Randomisation and blinding

Wards were allocated to intervention (*n* = 6) or control (*n* = 4) in an unequal allocation ratio (3:2) via minimisation using MinimPy [38]. This meant blinding was not possible. An unequal allocation ratio was chosen to ensure robust data for exploring feasibility and acceptability of the intervention across a representative range of specialities. Naïve minimisation was undertaken by a statistician at York Trials Unit (YTU) with a base probability 1.0 (i.e. deterministic minimisation) using three minimisation factors: ward speciality; percentage of patients aged 75 years and over, and NHS trust.

### Analysis

Feasibility outcomes, including the feasibility of conducting a full cost-effectiveness analysis in the definitive trial, were reported descriptively by trial arm. Ward and participant-level baseline data were reported using descriptive statistics. Continuous variables were summarised using the mean, standard deviation, median, and interquartile range (IQR). Categorical variables were summarised using a count and percentage. No formal statistical significance testing to test baseline imbalances between the treatment arms was undertaken. As this was a feasibility study, all analyses were exploratory, and between-group differences were not formally assessed. All analyses were conducted in Stata v.16 (StataCorp, College Station, Texas, USA).

### Qualitative evaluation of feasibility

The qualitative evaluation aimed to purposively recruit three to four patients/carers and four to five members of staff per intervention ward. Patients received up to £30 for participation.

Non-participant observations included intervention set up/facilitation, staff training and roles, introducing the YCNY booklet, staff and patient interactions, discharge, and relevant ward activities. Observations were captured via flexible but structured field notes.

Semi-structured patient interviews were conducted approximately 1 week post-discharge and, if patients continued to use the intervention at home, at 30-day post-discharge. Key intervention ward staff and a sample of control ward staff were interviewed to assess acceptability. Topic guides were informed by the COM-B framework [39]. Interviews were audio recorded, or detailed notes were taken.

Data were synthesised using pen portraits [40] — an analytic process used to bring together different types of data over a longitudinal timeframe to create focused accounts. In this study, pen portraits were used to describe implementation, patient and staff engagement, and patient experiences post-discharge for each ward. Pen portraits were then analysed using thematic analysis [41].

## Results

### Recruitment

#### Wards

**Fig. 1:**
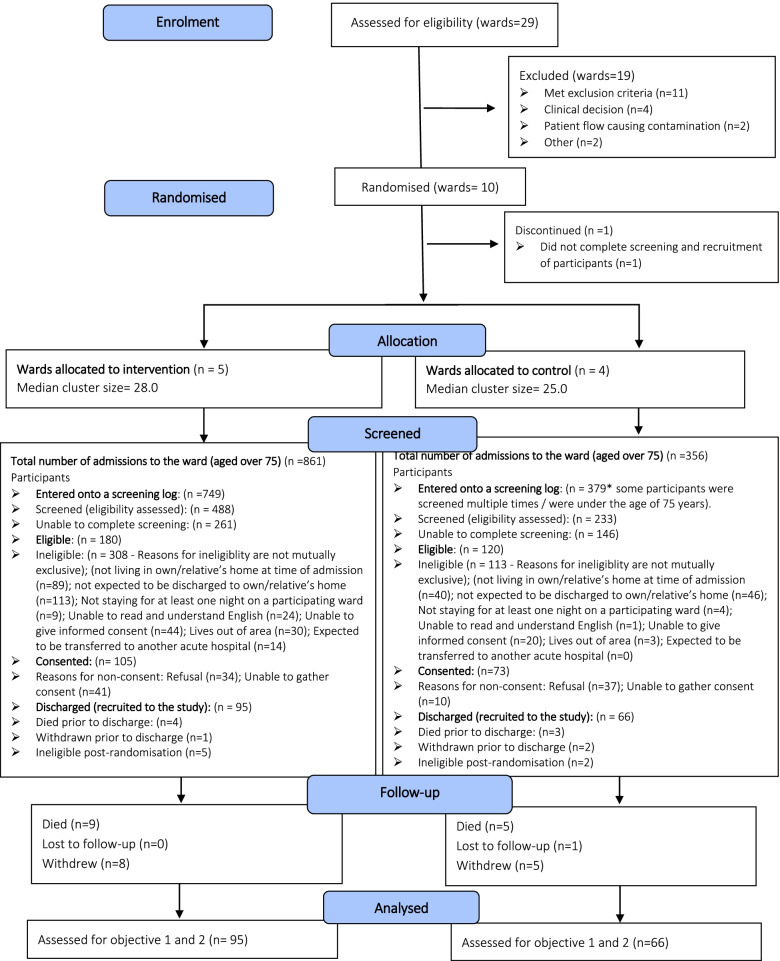
CONSORT style flow diagram showing ward and participant flow through the study

The flow of wards and participants through the trial is shown in Fig. 1. Ward specialities included older people’s medicine (*n* = 3), orthopaedic trauma (*n* = 3), stroke (*n* = 2), cardiology (*n* = 1), and intermediate care (*n* = 1).

Following randomisation, one NHS trust (Trust 1) which contained four participating wards could not start the trial within the planned timescales or deliver the intervention due to extreme staff shortages. Their wards were retained in the trial and treated according to their randomised allocation (intervention *n* = 2; control *n* = 2). One other intervention ward in Trust 2 withdrew prior to starting the trial due to a change in ward manager.

#### Patients

A total of 1128 patients (749 intervention, 379 control) were entered onto screening logs between December 2019 and March 2020. Screening data were complete for 721 (63.9%) patients. Of those successfully screened, 300 patients (180 intervention, 120 control) were deemed eligible and 421 (53.8%) were ineligible. Reasons for ineligibility are described in Fig. 1.

In total, 178 of the 300 eligible patients provided consent (105 intervention, 73 control). Of the remaining 122 eligible patients, 71 refused consent, and 51 could not consent for varying reasons (see Fig. 1). Seventeen patients became ineligible pre- (*n* = 10) or post-discharge (*n* = 7). Therefore, 161 participants (95 intervention, 66 control) were recruited to the study. Table 2 summarises the baseline characteristics of recruited participants.

**Table 2:** Baseline characteristics of participants

Variable		Intervention (***n*** = 95)	Control (***n*** = 66)	All (***n*** = 161)
Participant status	Patient	85 (89.5%)	56 (84.8%)	141 (87.6%)
	Informal carer	10 (10.5%)	10 (15.2%)	20 (12.4%)
Gender	Male	48 (50.5%)	18 (27.3%)	66 (41.0%)
	Female	47 (49.5%)	48 (72.7%)	95 (59.0%)
Age (years)	N	95 (100.0%)	66 (100.0%)	161 (100.0%)
	Mean (SD)	83.3 (5.2)	83.7 (4.8)	83.4 (5.0)
	Median (IQR)	83.0 (79, 87)	83.5 (81, 86)	83.0 (79, 87)
	Min, Max	75.0, 95.0	75.0, 95.0	75.0, 95.0
Ethnicity	White British	91 (95.8%)	66 (100.0%)	157 (97.5%)
	White Irish	2 (2.1%)	0 (0.0%)	2 (1.2%)
	White other	1 (1.1%)	0 (0.0%)	1 (0.6%)
	Pakistani	1 (1.1%)	0 (0.0%)	1 (0.6%)
Number of previous hospital admissions in the previous 12 months^a^	0	52 (54.7%)	30 (45.5%)	82 (50.9%)
	1	16 (16.8%)	18 (27.3%)	34 (21.1%)
	2	13 (13.7%)	12 (18.2%)	25 (15.5%)
	3	4 (4.2%)	0 (0.0%)	4 (2.5%)
	4+	5 (5.3%)	5 (7.6%)	10 (6.2%)
	Not reported	5 (5.3%)	1 (1.5%)	6 (3.7%)
Barthel index^a^	N	95 (100.0%)	66 (100.0%)	161 (100.0%)
	Mean (SD)	14.3 (4.3)	13.0 (4.5)	13.8 (4.4)
	Median (IQR)	15.0 (10, 18)	14.0 (10, 17)	15.0 (10, 17)
	Min, max	4, 20	1, 20	1, 20
Functional Comorbidity Index^a^	*N*	95 (100.0%)	66 (100.0%)	161 (100.0%)
	Mean (SD)	3.7 (2.1)	3.5 (2.0)	3.6 (2.0)
	Median (IQR)	3.0 (2, 5)	3.0 (2, 4)	3.0 (2, 5)
	Min, max	0, 10	0, 9	0, 10

^a^Clinical outcome measures were collected at baseline to assess the feasibility of data collection. Information on how the Barthel index and Functional Comorbidity Index are scored is provided in Table 1

### Retention

One-hundred forty-eight participants (91.9%: intervention = 87, control = 61) contributed routinely collected hospital readmissions data at 30-day post-discharge, which is the primary outcome measure for the definitive cRCT. Self-reported secondary outcome data were collected from 121 participants (75.2%: intervention = 72, control = 49) at the 5-day follow-up and from 95 participants (59%: intervention = 54, control = 41) at the 30-day follow-up. On occasion, the tight timescales between the first two follow-ups caused participant confusion. In conjunction with the Trial Management Group (TMG) and Trial Steering Committee (TSC), a decision was made to cease data collection early on 31st March 2020 due to the COVID-19 pandemic. Four participants had not reached the 30-day time point. Twenty-seven participants (16.8%, intervention = 11, control = 16) contributed to 90-day data, representing 57.4% of the 47 participants who had reached this time point. Following discharge, 28 participants discontinued, giving an attrition rate (post-discharge) of 17.4%. Reasons for discontinuation are summarised in Fig. 1.

### Outcomes

#### Routinely collected data

Routinely collected data were requested for participants who remained in the trial at the time of the data request. Readmission data up to 90-day post-discharge (or withdrawal/death) were successfully collected for 148 (91.9%) participants (Table 3). Routinely collected hospital readmission data were not collected for those (*n* = 13) who withdrew from the trial. Ward level baseline data were successfully collected across a 12-month period (supplementary file 2).

**Table 3:** Completion rates and scores for routinely collected and patient-reported outcome measures

Outcome measure	Intervention (*n* = 95)		Control (*n* = 66)		Overall (*n* = 161)	
	*n(%)*	Mean (SD)	*n(%)*	Mean (SD)	*n(%)*	Mean (SD)
Emergency readmissions						
30 days	87 (91.6%)	0.2 (0.4)	61 (92.4%)	0.2 (0.6)	148 (91.9%)	0.2 (0.5)
60 days	87 (91.6%)	0.3 (0.6)	61 (92.4%)	0.4 (0.7)	148 (91.9%)	0.3 (0.6)
90 days	87 (91.6%)	0.4 (0.6)	61 (92.4%)	0.5 (0.7)	148 (91.9%)	0.4 (0.7)
EQ5D-5L						
Baseline	95 (100.0%)	0.512 (0.3)	66 (100.0%)	0.441 (0.3)	161 (100.0%)	0.483 (0.3)
5 days	68 (71.6%)	0.495 (0.3)	48 (72.7%)	0.506 (0.3)	116 (72.0%)	0.499 (0.3)
30 days	51 (53.7%)	0.512 (0.3)	40 (60.6%)	0.484 (0.3)	91 (56.5%)	0.500 (0.3)
90 days	11 (11.6%)	0.416 (0.4)	16 (24.2%)	0.587 (0.3)	27 (16.8%)	0.517 (0.3)
EQ5D VAS						
Baseline	95 (100.0%)	50.1 (21.1)	66 (100.0%)	56.2 (20.0)	161 (100.0%)	52.6 (20.8)
5 days	69 (72.6%)	58.2 (18.0)	47 (71.2%)	53.7 (23.3)	116 (72.0%)	56.4 (20.3)
30 days	53 (55.8%)	60.5 (20.6)	39 (59.1%)	60.2 (20.0)	92 (57.1%)	60.4 (20.2)
90 days	11 (11.6%)	58.2 (20.3)	16 (24.2%)	67.8 (17.9)	27 (16.8%)	63.9 (19.1)
PACT-M						
5 days	62 (65.3%)	50.8 (9.2)	38 (57.6%)	49.5 (9.6)	100 (62.1%)	50.3 (9.3)
30 days	45 (47.4%)	54.4 (8.3)	34 (51.6%)	53.1 (9.0)	79 (49.1%)	53.8 (8.6)
90 days	9 (9.5%)	51.3 (8.3)	11 (16.7%)	57.5 (6.6)	20 (12.4%)	54.7 (7.9)
CTM-3						
5 days	63 (66.3%)	68.2 (18.6)	43 (65.2%)	64.0 (17.5)	106 (65.8%)	66.5 (18.2)
30 days	45 (47.4%)	67.5 (18.5)	33 (50%)	63.1 (17.0)	78 (48.4%)	65.7 (17.9)
90 days	11 (11.6%)	68.7 (19.8)	13 (19.7%)	63.2 (18.9)	24 (14.9%)	65.7 (19.1)

*NB* these measures were collected at follow-up to assess the feasibility of data collection. Information on how these measures are scored is provided in Table 1

Discharge destination data were categorised for 88 nonindividual consenting patients. Seventy-five (85%) were discharged to their own or a relative’s home suggesting an error rate of approximately 15% when relying on coded ‘usual place of residence’ for our primary outcome.

#### Patient-reported data

Completion of items within questionnaires was generally high and similar across groups, with little missing data. Full summaries of completion rates can be found in supplementary file 3.

### Contamination

Data pertaining to the movement of participants across wards during the study period were available for 148 (91.1%) participants with 44 of those participants (29.7%) having at least one ward move before discharge. Only one patient from an intervention ward moved to a control ward with none moving in the other direction. One-hundred four patients (70.3%: 57 intervention; 47 control) were discharged home from the recruiting ward.

No unexpected serious adverse events were identified.

### Health economics

No data were missing in the EQ5D at baseline (Table 3). EQ5D completion rates fell to 72% (71.6% intervention; 72.7% control) at 5 days, 56.5% at 30 days, and 17.0% at 90 days as the number of returned questionnaires declined and data collection ceased due to the pandemic. A similar pattern emerged in the resource use data. Completeness of items within measures was high for participants that returned the questionnaires (supplementary file 3).

### Qualitative evaluation

Three intervention wards (A, B, and C) were included in the qualitative evaluation. Two intervention wards were unable to deliver the intervention due to extreme staff shortages within their trust, and one intervention ward withdrew from the study completely due to a change in ward management. Data were gathered from ten patients, 17 multidisciplinary staff, and via 91 discrete ward-level observations. The acceptability and feasibility of the YCNY intervention and implementation strategy were summarised into six themes.

#### Acceptability and usability

In general, staff typically viewed YCNY as complementary to their work and agreed with the principle of patient involvement. Some nursing staff positively commented that YCNY reminded them of how nursing once was. Generally, the intervention was considered acceptable for all patients. Staff reported positive patient responses and felt that YCNY facilitated earlier conversations about discharge. Staff thought a patient-friendly discharge summary was needed.

Patients and carers mostly viewed the intervention favourably. Although some participants regarded the overall message to be ‘basic’, many still felt it would help patients who were less involved in their care. Other participants, however, favoured simplicity, with one participant suggesting reducing the number of questions to one per page. Some used the booklet to ask questions and take notes during their stay and reported that it legitimised asking questions, especially when staff appeared busy. One family member described how the booklet helped her think of questions to ask staff. Despite this, patients rarely used the YCNY booklet to communicate with staff directly (e.g. by propping up questions for staff to see).

#### Full implementation was difficult to achieve due to ward pressures

Despite initial enthusiasm, staff on wards A and B struggled to systematically deliver the YCNY booklet. On ward C, a highly organised discharge coordinator successfully delivered the YCNY booklet, but, without broader staff buy-in, patients received limited support to use it. Possibly as a consequence of staff pressures, some patients received the booklet close to discharge, therefore limiting its utility in hospital. Delivery of the YCNY film was hampered by a lack of technology. Staff also struggled to complete the patient-friendly care summary due to the following: time, fear of transcribing errors (especially into a patient-friendly language), concerns about providing advice on side effects and potentially vague symptoms, and a lack of information technology (IT) integration (the care summary was not embedded within the organisations’ electronic health records). Flexible staff-led responses to support the YCNY were sidelined other than on ward C, which advertised a patient exercise class.

#### Implementation must account for fluctuating patient health, capacity, and preferences

Some staff and patients shared concerns that the booklet could induce anxiety. A senior nurse questioned its appropriateness for some stroke patients early in their admission, while one patient requested that the booklet was explained to them when they felt up to it. This implies the need for patient introductions to be sensitive to daily fluctuating health states. Overall, providing support to use the booklet, timely introductions, and involvement of family were considered important.

#### Effective implementation requires sustained multidisciplinary engagement

Forming a functioning multidisciplinary team (MDT) around the intervention proved challenging. YCNY was more often delivered by individuals or one staff group who sometimes felt burdened. Likewise, ward managers had concerns about staff workload, and theirs and the research team’s efforts to broaden support were unsuccessful. Lack of clarity about supporting roles for clinical staff in implementing the intervention impacted on the ability of staff to support the flexible aspects of YCNY. Indeed, one patient specifically cited the apparent lack of buy-in from clinical staff as a reason for not fully engaging with the intervention. We observed an unsustained surge in activity on ward A due to the efforts of a senior clinician who encouraged involvement of the discharge team, doctors’ assistant, clinicians, and junior doctors.

#### Confusion about staff roles

Some staff were confused about their role beyond delivering the booklet, film, and care summary including, for example, who could support better patient communications about medications. Some staff felt that although the training sessions provided a clear rationale for patient involvement, they were not sufficiently task focused. Confusion existed over research processes versus delivery of the intervention. Some staff thought that researchers would support intervention delivery, while staff on ward B, where clinical trials were common, frequently confused implementing the intervention at a ward rather than patient level. As such, they incorrectly thought that the intervention could only be delivered to consented patents (rather than any patient on the wards for whom it may be clinically relevant).

#### Organisational contexts hindered implementation

Across participating trusts, there were a number of policies that hindered the implementation of the YCNY intervention. For example, a trust-wide limit on visiting hours restricted family involvement, and policies to mitigate the risks of falls and medication errors stymied efforts to encourage patients to mobilise or practise taking medications. Indirect or dispersed line management also sometimes made it difficult to involve staff. For example, one discharge coordinator, who was not line managed by the ward manager on the intervention ward, was told to prioritise their core work, despite the ward manager wanting to involve them in the intervention.

## Discussion

This study aimed to assess the feasibility of delivering the YCNY intervention and the trial methodology. We found that overall, the study design and associated trial procedures were feasible. Several methodological learning points were identified that will inform the delivery of a robust and efficient definitive trial. On the whole, the intervention was acceptable to staff and patients, although substantial implementation challenges were identified. The changes that will be made ahead of the definitive cluster randomised control trial are outlined in Table 4.

**Table 4:** Changes to the intervention and trial methodology ahead of a definitive cluster randomised controlled trial

Problem identified during the feasibility trial	Change made for the definitive trial
Attrition of wards and participants	Over recruit wards and increase the recruitment target to 25 patients per ward over a 5-month period
A relatively high margin of error when using routinely coded emergency readmission data as the primary outcome for our target patient population	We will seek permission from the Confidentiality Advisory Group (CAG) and Research Ethics Committee (REC) to check the actual discharge destinations of patients in the definitive trial via a nonindividual consent process
Attrition of self-reported data at 30- and 90-day follow-ups	We will make the data collection periods at 5 and 30 days more distinct. We will provide a supportive telephone call to all participants at each follow up
Booklet use was influenced by patients’ fluctuating capacity and health. The prop-up feature was rarely used	The language in the booklet will be simplified and its overall length reduced. The prop-up feature will be removed. Staff training will include greater emphasis on communication with patients and encouraging the booklets use
Limited delivery of the patient-friendly care summary	Care summary tailored at a ward rather than individual level to reduce associated risks. Distributed flexibly at a suitable time during the patients stay
Difficulty accessing the patient film	Intervention wards will be provided with a tablet to show the patient film
Managing risks associated with the flexible activities to enhance health, medicines, daily activities, and escalation	Engage a broader multidisciplinary team earlier during study set-up to explore what types of activities staff could engage with
Difficulties implementing and distributing the intervention	Greater emphasis on leadership and teamwork during setup. Explore the supporting role of volunteers and/or quality improvement teams

### Screening, recruitment, and retention

The withdrawal and/or inability to deliver YCNY on three wards is indicative of current NHS pressures [42, 43]. To mitigate against this in the definitive trial, we will over-recruit eligible wards to account for ward-level attrition. Similarly, due to attrition rates being higher than anticipated, the recruitment target will be increased in the definitive trial to 25 patients per ward over a 5-month period.

### Obtaining primary and secondary outcome data

Primary outcome data (emergency readmissions) were successfully extracted for almost all consented participants. However, the relatively high margin of error associated with using routinely coded data to inform our primary outcome for our target patient population means we will seek permission to check the actual discharge destinations of patients in the definitive trial (via a nonindividual consent process).

To reduce attrition of self-reported secondary outcome and health economic data at 30 and 90 days, a greater gap will be left between the last reminder contact that is made for the 5-day follow-up and the first contact that is made for the 30-day follow-up. By making the follow-up periods more distinct, we hope to reduce participant confusion about whether the questionnaires need to be returned or not. Furthermore, all participants will receive supportive telephone calls at all time points with a view to increasing response rates [44, 45].

### Contamination and fidelity

No changes will be made to how wards are identified in the definitive trial as almost no contamination occurred between control and intervention wards. However, approximately one-quarter of patients were not discharged from their recruiting ward suggesting that, even if implementation of the care summary had not been an issue, a proportion of patients on intervention wards are unlikely to have received the patient patient-friendly care summary simply because of where they were discharged from. In the definitive trial, wards will be encouraged to deliver the care summary at a time that suits their patients and ward routines rather than mandating its delivery at discharge. It is hoped that this more flexible approach will improve intervention fidelity and support implementation.

### Acceptability, usability, and feasibility of YCNY and its implementation

To ensure that patients can access the film, we will provide tablets in the definitive trial. Furthermore, as use of the booklet was influenced by fluctuations in patients’ health, capacity, and needs, some of its language will be simplified and its overall length reduced. It is known that patients are often unwilling to ask questions particularly if they have not been invited to do so by staff [46]. In this study, patients tended to refer to the booklet privately, rather than propping it up to display questions for staff. This observations may represent ‘compliant noninvolvement’, whereby patients receive care without questioning it and/or are passive when receiving or seeking information from staff [47]. As patients did not use the booklet as a visual prompt, and few staff encouraged it to be used in this way, the costly prop-up feature will be removed. However, findings suggest that efforts to enhance the culture of communication between staff and patients in the definitive trial will be critical. During staff training within the definitive trial, the importance of verbal and non-verbal communication with patients will be emphasised, and staff will be encouraged to reflect on current communication practices and ways in which they could enhance communication to support patients to interact with the intervention and be more involved in their care.

The inadequacy of discharge letters for both staff and patients is well recognised [48], and the various challenges staff faced in implementing the patient-friendly care summary are not unique [49]. Some represent systemic issues that cannot be addressed within the scope of our definitive trial, for example, changing each organisation’s electronic health record to incorporate the patient-friendly care summary so that individualised information can be given to patients at discharge. To ensure that some patient-friendly information is provided to support patients post-discharge, the care summary will be simplified in the definitive trial and replaced by a document which is tailored at a ward rather than individual level, thus reducing the time and risks associated with staff completing it.

Challenges relating to risk management were also raised in relation to the flexible activities staff could chose to enhance the four activities (health, medicines, daily activities, and escalation). For example, staff expressed concern about the risks and policies associated with supporting patients to mobilise or self-medicate in hospital. If risk cannot be shared across system boundaries, similar transitional care interventions and improvement work is unlikely to succeed. Policy, procedure, and cultural changes are required so that risk can be ‘held’ more effectively across the system rather than being managed within specific organisations and teams (wards or professional groups) where pressures and priorities do not necessarily match those of the patient. While setting up the definitive trial at a trust and ward level, researchers will seek to engage a broader multidisciplinary team (e.g. matrons and pharmacists) to explore activities that would be acceptable, and staff might feel comfortable, to implement.

Many implementation challenges were encountered including lack of leadership and teamwork to develop a sustainable action plan. In the definitive trial, leadership and teamwork will be encouraged earlier on in the set-up process so that they can play a galvanising role in engaging and sustaining ward-level involvement. Where diverse ward specialties are involved, the support of multiple leaders may be necessary.

Distributing the fixed intervention components was challenging for staff, in part due to competing tasks. In the definitive trial, non-ward staff, such as volunteers or quality improvement teams, could be engaged to support these basic tasks, thus freeing up ward staff to concentrate on encouraging patients to use them during their hospital stay and delivering the flexible staff, facing intervention components. Volunteers have previously supported the implementation of a patient involvement intervention [50]. However, as they are not regular members of the ward team, an embedding period would be required to establish the processes and support required for them to successfully complete these tasks.

### Limitations

A key limitation of this trial is that the scope and depth of the qualitative evaluation was greatly reduced by the full withdrawal and/or inability of three wards to deliver the intervention. Although data represented the ward specialties that will be included in the definitive trial, this will have limited our learning about the intervention and its implementation. Additionally, participants almost exclusively identified as being White British and so we are unable to say how feasible the intervention and/or trial procedures are to people from different ethnic backgrounds.

The trial was conducted prior to the COVID-19 pandemic. Although many of the trial procedures (e.g. ability to conduct follow-up and collect routine outcome data) are unlikely to be affected, changes to how patient care is delivered (e.g. reorganisation of ward specialties, quicker patient discharges, and restricted visiting), and increased pressures experienced by ward staff, may impact how we identify eligible wards and the implementation of YCNY during the definitive trial.

## Conclusion

YCNY is an innovative intervention which aims to enhance the involvement of older people during transitions of care. By supporting patients to know more and do more in hospital, it is anticipated that they will be better prepared to manage their care safely at home, thereby enhancing system resilience. Implementing YCNY is likely to require a change in ward culture to enhance communication and support patients to mobilise and practice taking their medications. Staff need to be supported to accept and manage risk within the hospital setting to reduce risk for patients once they are home. This feasibility trial highlighted the importance of engaging senior management to support this culture change and identified a number of systemic pressures that hindered implementation of both the fixed and flexible intervention components. If these implementation challenges can be overcome, YCNY represents a step towards involving patients as true partners in their own care. A definitive trial is required to assess the effectiveness of YCNY in enhancing safety and experience during transitions from hospital to home.

## Supplementary Information

**Additional file 1: Supplementary file 1**. CONSORT 2010 checklist of information to include when reporting a pilot or feasibility trial.

**Additional file 2: Supplementary file 2**: Summary of ward level baseline data over the previous 12 month period

**Additional file 3: Supplementary file 3**: Completion rates. **Table 1**: Data completeness of outcome measures collected at baseline. **Table 2**: Summary of data completeness for measures included in the T1 assessment for those who have completed a T1 questionnaire*.***Table 3**: Summary of data completeness for measures included in the T2 assessment for those who have completed a T2 questionnaire*.***Table 4**: Summary of data completeness for measures included in the T3 assessment for those who have completed a T3 questionnaire.

## Data Availability

Requests to access the PACT data should be made to the corresponding author and will be considered on a case-by-case basis by the Chief Investigator and Trial Management Group. All data requests for quantitative data will be managed in accordance with YTU, University of York, processes and procedures.

## Abbreviations

CAG: Confidentiality Advisory Group
CONSORT: Consolidated Standards of Reporting Trials
cRCT: Cluster randomised controlled trial
CSRI: Client Service Receipt Inventory
CTM: Care Transitions Measure
IT: Information technology
MDT: Multidisciplinary team
NIHR: National Institute for Health Research
PACT: Partners at Care Transitions
RCT: Randomised controlled trial
QALYs: Quality-adjusted life years
TMG: Trial Management Group
TSC: Trial Steering Committee
YCNY: Your Care Needs You
YQSR: Yorkshire Quality and Safety Research Group
YTU: York Trials Unit
