# Supplementary material for: Improving the safety and experience of transitions from hospital to home: a cluster randomised controlled feasibility trial of the 'Your Care Needs You' intervention versus usual care
Source: Pilot Feasibility Stud. 2022 Oct 1;8:222. doi: 10.1186/s40814-022-01180-3 (PMC9525931; doi:10.1186/s40814-022-01180-3)
Supplement: Supplementary file 2 — Additional file 2: Supplementary file 2: Summary of ward level baseline data over the previous 12 month period [file 40814_2022_1180_MOESM2_ESM.docx]

| **Ward** | **Readmissions within 30-days among patients who are discharged**  **<75, n (%)** | **Readmissions within 30-days among patients who are discharged**  **≥75, n (%)** | **Average length of hospital stay (days) who are admitted to a participating ward <75, n (%)** | **Average length of hospital stay (days) who are admitted to a participating ward ≥75, n (%)** |
| --- | --- | --- | --- | --- |
| **Ward 10** | 68 (8.5%) | 73 (11.6%) | 4.9 | 7.9 |
| **Ward 11** | 19 (19.4%) | 93 (28.7%) | 20.8 | 20.0 |
| **Ward 12** | 5 (9.4%) | 104 (17.2%) | 9.9 | 11.6 |
| **Ward 20** | 64 (18.5%) | 45 (12.7%) | 19.8 | 18.6 |
| **Ward 21** | 22 (10.3%) | 29 (11.6%) | 11.8 | 22.1 |
| **Ward 30** | 9 (6.8%) | 16 (8.7%) | 32.3 | 24.4 |
| **Ward 31** | 11 (24.4%) | 137 (16.3%) | 15.7 | 15.8 |
| **Ward 32** | 11 (12.4%) | 112 (17.5%) | 12.3 | 17.8 |
| **Ward 33** | 32 (8.0%) | 53 (13.7%) | 7.0 | 21.0 |

**Supplementary file 2: Summary of ward level baseline data over the previous 12 month period**
