# Supplementary material for: Improving the safety and experience of transitions from hospital to home: a cluster randomised controlled feasibility trial of the 'Your Care Needs You' intervention versus usual care
Source: Pilot Feasibility Stud. 2022 Oct 1;8:222. doi: 10.1186/s40814-022-01180-3 (PMC9525931; doi:10.1186/s40814-022-01180-3)
Supplement: Supplementary file 3 — Additional file 3: Supplementary file 3: Completion rates. Table 1: Data completeness of outcome measures collected at baseline. Table 2: Summary of data completeness for measures included in the T1 assessment for those who have completed a T1 questionnaire.Table 3: Summary of data completeness for measures included in the T2 assessment for those who have completed a T2 questionnaire.Table 4: Summary of data completeness for measures included in the T3 assessment for those who have completed a T3 questionnaire. [file 40814_2022_1180_MOESM3_ESM.docx]

**Supplementary file 3: Completion rates**

**Table 1: Data completeness of outcome measures collected at baseline**

|  | Intervention (N=95) | Usual Care (N=66) | Total  (N=161) |
| --- | --- | --- | --- |
| **Baseline EQ5D-5L (5 item questionnaire)** |  |  |  |
| No missing items (5 completed) | 95 (100%) | 66 (100%) | 161 (100%) |
| 1 or more missing item (cannot be scored) | 0 (0%) | 0 (0%) | 0 (0%) |
| **EQ5D Visual Analogue Scale (1 item)** |  |  |  |
| EQ5D VAS completed | 95 (100%) | 66 (100%) | 161 (100%) |
| EQ5D VAS not completed | 0 (0%) | 0 (0%) | 0 (0%) |
| **Barthel Index (10 item questionnaire)** |  |  |  |
| No missing items (10 completed) | 87 (91.6%) | 65 (98.5%) | 152 (94.4%) |
| 1 missing item (9 completed) | 7 (7.4%) | 1 (1.5%) | 8 (5.0%) |
| 2 missing items (8 completed) | 1 (1.1%) | 0 (0.0%) | 1 (0.6%) |
| More than 2 missing items | 0 (0.0%) | 0 (0.0%) | 0 (0.0%) |
| **Functional Co-morbidity Index (18 item questionnaire)** |  |  |  |
| Completed at least one item | 93 (97.9%) | 62 (93.9%) | 155 (96.3%) |
| No items completed | 2 (2.1%) | 4 (6.1%) | 6 (3.7%) |

**Table 2: Summary of data completeness for measures included in the T1 assessment for those who have completed a T1 questionnaire**

|  | Intervention  (n = 95) | Control  (n = 66) | Total  (n = 161) |
| --- | --- | --- | --- |
| **T1 Questionnaire Status** |  |  |  |
| Completed | 72 (75.8%) | 49 (74.2%) | 121 (75.2%) |
| Not completed | 23 (24.2%) | 17 (25.8%) | 40 (33.1%) |
| **EQ5D-5L (5 item questionnaire)** |  |  |  |
| No missing items (5 completed) | 68 (94.4%) | 48 (98.0%) | 116 (95.9%) |
| 1 or more missing item (cannot be scored) | 4 (5.6%) | 1 (2.0%) | 5 (4.1%) |
| **EQ5D Visual Analogue Scale (1 item)** |  |  |  |
| EQ5D VAS completed | 69 (95.8%) | 47 (95.9%) | 116 (95.9%) |
| EQ5D VAS not completed | 3 (4.2%) | 2 (4.1%) | 5 (4.1%) |
| **Care Transitions Measure (3 items)** |  |  |  |
| No missing items (3 completed) | 63 (87.5%) | 43 (87.8%) | 106 (87.6%) |
| 1 or more missing item | 2 (2.8%) | 0 (0.0%) | 2 (1.7%) |
| Carer questionnaire (CTM-3 not included) | 7 (9.7%) | 6 (12.2%) | 13 (10.7%) |
| **Patient At Care Transitions Measure (PACT-M) (8 items)** |  |  |  |
| No missing items (8 completed) | 64 (88.9%) | 41 (83.7%) | 105 (86.8%) |
| 1 missing item | 0 (0.0%) | 0 (0.0%) | 0 (0.0%) |
| 2 missing items | 1 (1.4%) | 0 (0.0%) | 1 (0.8%) |
| 3 or more missing items | 0 (0.0%) | 2 (4.1%) | 2 (1.7%) |
| Carer questionnaire (PACT-M not included) | 7 (9.7%) | 6 (12.2%) | 13 (10.7%) |
| **Patient At Care Transitions Measure (PACT-M) adverse events (7 items)** |  |  |  |
| No missing items (7 completed) | 66 (91.7%) | 40 (81.6%) | 106 (87.6%) |
| 1 missing item | 4 (5.6%) | 6 (12.2%) | 10 (8.3%) |
| 2 missing items | 1 (1.4%) | 2 (4.1%) | 3 (2.5%) |
| 3 or more missing items | 1 (1.4%) | 1 (2.0%) | 2 (1.7%) |
| **Utility of the Intervention** |  |  |  |
| **Did you receive a ‘YCNY’ booklet?** |  |  |  |
| Completed | 66 (91.7%) | 49 (100.0%) | 115 (95.0%) |
| Not completed | 6 (8.3%) | 0 (0.0%) | 6 (5.0%) |
| **Were you given a ‘YCNY’ care summary?** |  |  |  |
| Completed | 65 (90.3%) | 47 (95.9%) | 112 (92.6%) |
| Not completed | 7 (9.7%) | 2 (4.1%) | 9 (7.4%) |

**Table 3: Summary of data completeness for measures included in the T2 assessment for those who have completed a T2 questionnaire**

|  | Intervention  (n = 95) | Control  (n = 66) | Total  (n = 161) |
| --- | --- | --- | --- |
| **T2 questionnaire status** |  |  |  |
| Completed | 54 (56.8%) | 41 (62.1%) | 95 (59.0%) |
| Not completed | 41 (43.2%) | 25 (37.9%) | 66 (41.0%) |
| **EQ5D-5L (5 item questionnaire)** |  |  |  |
| No missing items (5 completed) | 51 (94.4%) | 40 (97.6%) | 91 (95.8%) |
| 1 or more missing item (cannot be scored) | 3 (5.6%) | 1 (2.4%) | 4 (4.2%) |
| **EQ5D Visual Analogue Scale (1 item)** |  |  |  |
| EQ5D VAS completed | 53 (98.1%) | 38 (92.7%) | 92 (96.8%) |
| EQ5D VAS not completed | 1 (1.9%) | 2 (4.9%) | 3 (3.2%) |
| **Care Transitions Measure (3 items)** |  |  |  |
| No missing items (3 completed) | 45 (83.3%) | 35 (85.4%) | 80 (84.2%) |
| 1 or more missing item | 3 (5.6%) | 1 (2.4%) | 4 (4.2%) |
| Carer questionnaire (CTM-3 not included) | 6 (11.1%) | 5 (12.2%) | 11 (11.6%) |
| **Patient At Care Transitions Measure (PACT-M) (8 items)** |  |  |  |
| No missing items (8 completed) | 45 (83.3%) | 34 (82.9%) | 79 (83.2%) |
| 1 missing item | 1 (1.9%) | 0 (0.0%) | 1 (1.1%) |
| 2 missing items | 1 (1.9%) | 0 (0.0%) | 1 (1.1%) |
| 3 or more missing items | 1 (1.9%) | 2 (4.9%) | 3 (3.2%) |
| Carer questionnaire (PACT-M not included) | 6 (11.1%) | 5 (12.2%) | 11 (11.6%) |
| **Patient At Care Transitions Measure (PACT-M) adverse events (7 items)** |  |  |  |
| No missing items (7 completed) | 52 (96.3%) | 39 (95.1%) | 91 (95.8%) |
| 1 missing item | 1 (1.9%) | 2 (4.9%) | 3 (3.2%) |
| 2 missing items | 0 (0.0%) | 0 (0.0%) | 0 (0.0%) |
| 3 or more missing items | 1 (1.9%) | 0 (0.0%) | 1 (1.1%) |
| **Utility of the Intervention** |  |  |  |
| **Did you receive a ‘YCNY’ booklet?** |  |  |  |
| Completed | 49 (90.7%) | 39 (95.1%) | 88 (92.6%) |
| Not completed | 5 (9.3%) | 2 (4.9%) | 7 (7.4%) |
| **Were you given a ‘YCNY’ care summary?** |  |  |  |
| Completed | 46 (85.2%) | 40 (97.6%) | 86 (90.5%) |
| Not completed | 8 (14.8%) | 1 (2.4%) | 9 (9.5%) |

**Table 4: Summary of data completeness for measures included in the T3 assessment for those who have completed a T3 questionnaire**

|  | Intervention  (n = 95) | Control  (n =66) | Total  (n = 161) |
| --- | --- | --- | --- |
| **T3 questionnaire status** |  |  |  |
| Completed | 11 (11.6%) | 16 (24.2%) | 27 (14.9%) |
| Not completed | 84 (88.4%) | 50 (75.8%) | 3 (1.9%) |
| **EQ5D-5L (5 item questionnaire)** |  |  |  |
| No missing items (5 completed) | 11 (100.0%) | 16 (100.0%) | 27 (100.0%) |
| 1 or more missing item (cannot be scored) | 0 (0.0%) | 0 (0.0%) | 0 (0.0%) |
| **EQ5D Visual Analogue Scale (1 item)** |  |  |  |
| EQ5D VAS completed | 11 (100.0%) | 16 (100.0%) | 27 (100.0%) |
| EQ5D VAS not completed | 0 (0.0%) | 0 (0.0%) | 0 (0.0%) |
| **Care Transitions Measure (3 items)** |  |  |  |
| No missing items (3 completed) | 11 (100.0%) | 13 (81.3%) | 24 (88.9%) |
| 1 or more missing item | 0 (0.0%) | 0 (0.0%) | 0 (0.0%) |
| Carer questionnaire (CTM-3 not included) | 0 (0.0%) | 3 (18.8%) | 3 (11.1%) |
| **Patient At Care Transitions Measure (PACT-M) (8 items)** |  |  |  |
| No missing items (8 completed) | 9 (81.8%) | 10 (62.5%) | 19 (70.4%) |
| 1 missing item | 0 (0.0%) | 1 (6.3%) | 1 (3.7%) |
| 2 missing items | 0 (0.0%) | 1 (6.3%) | 1 (3.7%) |
| 3 or more missing items | 2 (18.2%) | 1 (6.3%) | 3 (11.1%) |
| Carer questionnaire (PACT-M not included) | 0 (0.0%) | 3 (18.8%) | 3 (11.1%) |
| **Patient At Care Transitions Measure (PACT-M) adverse events (7 items)** |  |  |  |
| No missing items (7 completed) | 9 (81.8%) | 16 (100.0%) | 25 (92.6%) |
| 1 missing item | 0 (0.0%) | 0 (0.0%) | 0 (0.0%) |
| 2 missing items | 0 (0.0%) | 0 (0.0%) | 0 (0.0%) |
| 3 or more missing items | 2 (18.2%) | 0 (0.0%) | 2 (7.4%) |
